# Dual phosphorylation of Sin1 at T86 and T398 negatively regulates mTORC2 complex integrity and activity

**DOI:** 10.1007/s13238-014-0021-8

**Published:** 2014-01-31

**Authors:** Pengda Liu, Jianping Guo, Wenjian Gan, Wenyi Wei

**Affiliations:** Department of Pathology, Beth Israel Deaconess Medical Center, Harvard Medical School, Boston, MA 02115 USA

## Abstract

Mammalian target of rapamycin (mTOR) plays essential roles in cell proliferation, survival and metabolism by forming at least two functional distinct multi-protein complexes, mTORC1 and mTORC2. External growth signals can be received and interpreted by mTORC2 and further transduced to mTORC1. On the other hand, mTORC1 can sense inner-cellular physiological cues such as amino acids and energy states and can indirectly suppress mTORC2 activity in part through phosphorylation of its upstream adaptors, IRS-1 or Grb10, under insulin or IGF-1 stimulation conditions. To date, upstream signaling pathways governing mTORC1 activation have been studied extensively, while the mechanisms modulating mTORC2 activity remain largely elusive. We recently reported that Sin1, an essential mTORC2 subunit, was phosphorylated by either Akt or S6K in a cellular context-dependent manner. More importantly, phosphorylation of Sin1 at T86 and T398 led to a dissociation of Sin1 from the functional mTORC2 holo-enzyme, resulting in reduced Akt activity and sensitizing cells to various apoptotic challenges. Notably, an ovarian cancer patient-derived Sin1-R81T mutation abolished Sin1-T86 phosphorylation by disrupting the canonical S6K-phoshorylation motif, thereby bypassing Sin1-phosphorylation-mediated suppression of mTORC2 and leading to sustained Akt signaling to promote tumorigenesis. Our work therefore provided physiological and pathological evidence to reveal the biological significance of Sin1 phosphorylation-mediated suppression of the mTOR/Akt oncogenic signaling, and further suggested that misregulation of this process might contribute to Akt hyper-activation that is frequently observed in human cancers.

## mTORC1 and mTORC2 are functionally distinct mTOR-containing protein kinase complexes

Mammalian target of rapamycin (mTOR) is a highly conserved protein kinase (van Dam et al., 2011) belonging to the phosphatidylinositol-3-kinase-related protein kinase (PIKK) super-family. Biologically, mTOR is a master regulator of cellular homeostasis, cell growth and proliferation as well as metabolism in a broad range of physiological (Wullschleger et al., 2006; Zoncu et al., 2011b) and pathological settings, including diabetes and cancer (Guertin and Sabatini, 2007; Inoki et al., 2005). In doing so, mTOR serves as a central sensor for cellular physiological cues such as growth signals and nutrients, by functioning as an essential catalytic subunit in two functionally distinct complexes, mTOR complex 1 (mTORC1) (Kim et al., 2002) and mTOR complex 2 (mTORC2) (See (Sabatini, 2006) and (Wullschleger et al., 2006) for review). These two complexes are distinguished by their accessory protein composition, localization and function. Specifically, both mTORC1 and mTORC2 share the common components mTOR and GβL/mLST8, while the unique subunit Raptor defines mTORC1 (Kim et al., 2002) whereas Rictor (Sarbassov et al., 2004) and Sin1 (Frias et al., 2006; Guertin et al., 2006; Jacinto et al., 2006; Wullschleger et al., 2005; Yang et al., 2006) define mTORC2. mTORC1 was reported to localize on the outer membrane of lysosome, which is critical for its activation and physiological function (Bar-Peled and Sabatini, 2012; Thoreen et al., 2012), while mTORC2 association with ribosomes was recently demonstrated to be critical for mTORC2 activity (Zinzalla et al., 2011).

In echoing their unique subcellular localizations, mTORC1 and mTORC2 display distinct cellular functions by targeting different sets of downstream effectors for phosphorylation and function. Notably, mTORC1 promotes cap-dependent mRNA translation (Thoreen et al., 2012) and protein synthesis through direct phosphorylation and activation of its *bona fide* substrates S6K1, 4EBP1 and TFEB1 (Ma and Blenis, 2009; Pena-Llopis et al., 2011), inhibits autophagy through phosphorylation of Ulk1 (Chan, 2009) and establishes Treg-cell function by facilitating cholesterol and lipid metabolism through yet undefined phosphorylation targets (Zeng et al., 2013). On the other hand, mTORC2 was firstly identified to regulate cellular skeletal organization (Jacinto et al., 2004) and later shown to be indispensible in governing cell growth, proliferation, survival and anabolism, which are mainly through direct phosphorylation and activation of its physiological downstream targets including Akt (S473) (Sarbassov et al., 2005), SGK (S422) (Garcia-Martinez and Alessi, 2008) and PKCα (Ikenoue et al., 2008). Moreover, as mTOR serves as an oncogenic pathway to promote cellular growth and survival, deregulation of many components of the mTOR pathway has been implicated in human cancer and metabolic diseases (Weber and Gutmann, 2012; Zoncu et al., 2011b).

## mTORC1 and mTORC2 are differentially regulated in cells

Previous work clearly established that both activation of mTORC1 and mTORC2 are tightly, yet differentially controlled (Laplante and Sabatini, 2012; Weber and Gutmann, 2012). Mechanistically, when stimulated by extra-cellular growth signals, mTORC2 receives activation signals from the Ras/PI3K signaling pathway through undefined mechanisms, and activates mTORC1 by Akt-dependent phosphorylation of TSC2 (Inoki et al., 2002; Manning et al., 2002) or PRAS40 (Vander Haar et al., 2007), releasing their repression on mTORC1. In addition, mTORC1 can also sense cellular energy states and amino acid levels independent of mTORC2. Specifically, when cellular AMP levels are high, which indicates low energy status in cells, the LKB1/AMPK (AMP-activated serine/threonine protein kinase) pathway becomes activated, leading to inhibition of mTORC1 (Shaw, 2009), either by phosphorylation of TSC2 (Inoki et al., 2003) or by phosphorylation of the essential mTORC1 component Raptor (Gwinn et al., 2008). Furthermore, mTORC1, but not mTORC2, serves as an inner cellular amino acid sensor (Efeyan et al., 2012) dependent on its recruitment to lysosome surface by Rag (Sancak et al., 2010; Sancak et al., 2008), Ragulator (Bar-Peled et al., 2012) and the lysosome vacuolar H^+^-ATPase (Zoncu et al., 2011a) through an inside-out mechanism to control the timely activation of mTORC1.

The differential regulatory mechanisms of these two mTOR-containing complexes extend further to their altered responses to rapamycin. mTORC1 is sensitive to low-dose rapamycin treatment in both cell culture and mouse models, where rapamycin directly binds to FKBP12 and disrupts the interaction between Raptor and mTOR, suppressing mTORC1 assembly and activation (Oshiro et al., 2004; Yip et al., 2010). On the other hand, mTORC2 only responds to prolonged and chronic rapamycin treatment, in part because rapamycin cannot directly interfere with existing mTORC2 complex, but rather only blocks the assembly of mTORC2 from newly synthesized Rictor and mTOR (Sarbassov et al., 2006).

More importantly, mTORC1 could also be activated independent of mTORC2. Mice with deleted *S6K1* display elevated resistance to high-fat diet and weight gain, in part due to deficiency in adipocytes generation (Carnevalli et al., 2010*).* Adipose specific *Raptor* knockout mice phenocopied the *S6K1* knockout mice with regard to adipocyte generation (Polak et al., 2008), highlighting the critical role of mTORC1 in adipogenesis *in vivo*. However, *Rictor* knockout mice showed no defects in adipogenesis (Kumar et al., 2010), although an mTORC2 substrate, BTSA (a BSD domain-containing protein) has been characterized to be indispensible for adipogenesis (Kumar et al., 2010). Taken together, mTORC1 and mTORC2 could function synergistically or independently to maintain cell physiology.

## mTORC1 negatively regulates mTORC2 activation through indirect mechanisms mediated by phosphorylation of IRS-1 and GRB10

mTORC1 has also been shown to indirectly suppress mTORC2 signaling that in *TSC2*^*-/-*^ cells with elevated mTORC1/S6K activity, Akt activation was significantly reduced (Manning et al., 2005). Further studies revealed that elevated mTORC1/S6K could suppress the activation of the PI3K pathway through multiple negative feedback loops (Gual et al., 2005). Specifically, mTORC1 and S6K can phosphorylate IRS-1 to block its interaction with the p85 regulatory subunit of PI3K to negatively regulate the insulin-signaling pathway (Gual et al., 2005). More recently, both the Blennis (Yu et al., 2011) and Sabatini (Hsu et al., 2011) groups independently showed that Grb10 is phosphorylated and stabilized by mTORC1 to block the PI3K signaling. However, in response to certain stimuli such as PDGF and EGF, activation of mTORC2/Akt was not affected by depletion of Grb10, suggesting the presence of an additional mechanism for mTORC1 to suppress mTORC2. As genetic disruption of either *Rictor* or *Sin1* results in the inactivation of mTORC2 (Guertin et al., 2006; Jacinto et al., 2006), we reasoned that mTORC1/S6K-dependent regulation of mTORC2 might occur through these unique mTORC2 components in a direct manner. However, we and others previously showed that AGC kinases-mediated phosphorylation of Rictor at T1135 does not significantly affect mTORC2 complex integrity and its kinase activity (Dibble et al., 2009; Gao et al., 2010), which urged us to further examine whether the other mTORC2 essential component, Sin1, is a major target to mediate mTORC1/S6K’s negative regulation of mTORC2.

## mTORC1 negatively regulates mTORC2 activation through direct mechanisms mediated by phosphorylation of SIN1

**Figure 1:**
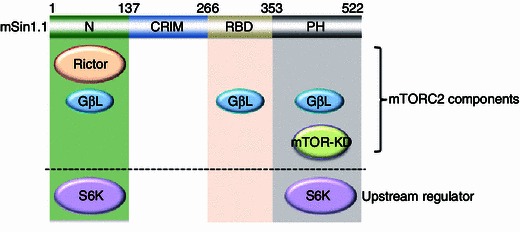
mTORC2 components and S6K interact with different domains of Sin1

**Figure 2:**
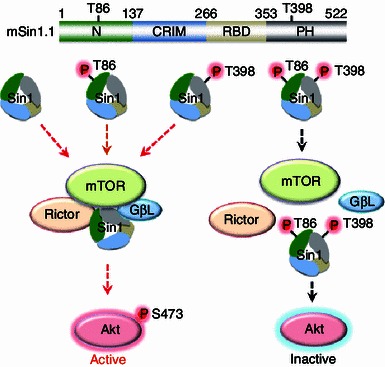
Phosphorylation of Sin1 at both T86 and T398 sites dissociates Sin1 from functional mTORC2 complex and subsequently suppresses Akt activation

To test if modulation of Sin1 is important for mTORC2 activity, we and others tested if Sin1 could be targeted for phosphorylation by Akt or S6K at either T86 and T398, or both sites, in adipocytes (Humphrey et al., 2013) or epithelial cells (Liu et al., 2013), respectively. Importantly, we found that phosphorylation of Sin1 on both the T86 and T398 sites led to a disassociation of Sin1 from other mTORC2 components, revealing a direct negative regulatory mechanism for mTORC2 governed by mTORC1 (Liu et al., 2013). Mechanistically, we observed that Sin1 utilized various domains to interact with mTORC2 components (Fig. 1), with its N-terminus binding to Rictor and GβL, RBD (Ras binding domain) binding to GβL, and the PH domain interacting with GβL and mTOR-KD (kinase domain). Interestingly, only the N and PH domains were capable of interacting with S6K1 (Fig. 1). Considering that the identified T86 and T398 phosphorylation sites are located in the N- and PH domains, respectively, it is therefore plausible that S6K could directly phosphorylate these two sites to influence mTORC2 integrity. In keeping with this notion, phosphorylation at T86 interfered with Sin1-N-terminus binding to Rictor, while phosphorylation at T398 impaired Sin1-PH domain interaction with the mTOR kinase domain. Importantly, Sin1 dissociation from the mTORC2 complex requires both phosphorylation events (Fig. 2). In addition, we demonstrated that Sin1 phosphorylation at both sites occurred in response to various external cellular stimuli including insulin, IGF1, PDGF and EGF, which significantly blocked the activation of Akt induced by these stimuli, proposing a novel negative feedback loop independent of IRS-1 and Grb10 to suppress mTORC2.

Physiologically, we demonstrated that Sin1 phosphorylation-mediated mTORC2 suppression on Akt led to increased levels of cleaved caspase 3 and subsequently sensitized cells to apoptosis-initiating agents such as etoposide or cisplatin. More importantly, we found that in freshly isolated splenic B cells, rapamycin treatment significantly reduced Sin1-T86 phosphorylation accompanied by an increase in Akt-S473 phosphorylation. A similar inverse correlation between phosphorylation of Sin1-T86 and Akt-S473 was also observed in mouse liver lysates, and rapamycin administration to whole mice led to reduced Sin1-pT86 concomitant with increased Akt-pS473 signals, further confirming the physiological significance of mTORC1-mediated negative regulation on mTORC2 function.

**Figure 3:**
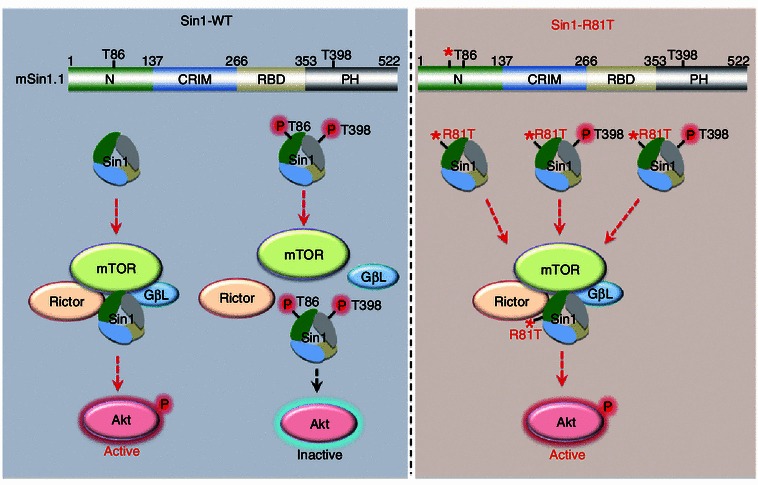
The cancer patient-derived Sin1-R81T mutation leads to stabilized mTORC2 integrity and sustained Akt activation to facilitate tumorigenesis

Pathologically, we screened a panel of human ovarian patient clinical samples by IHC and an inverse correlation between Sin1-pT86 and Akt-pS473 was observed in a certain number of cases. However, due to the limited number of patient samples available, the inverse correlation did not reach statistical significance, which warrants further larger-scale examinations. This inverse correlation was also observed in a panel of T-ALL cancer cell lines. Interestingly, two Sin1 mutations (R81T and S84L) were identified in ovarian cancer patients. Using biochemical assays we demonstrated that both of these mutations led to significantly reduced Sin1 phosphorylation at the T86 site, as these mutations impair the canonical AGC kinase consensus recognition motif “RxRxxpS/pT” (Alessi et al., 1996; Manning and Cantley, 2007). As phosphorylation at both T86 and T398 is necessary to dissociate Sin1 from the functional mTORC2 complex, substitutions at R81T or S84L diminished the possibility of co-occurrence of pT86 and pT398, resulting in stabilized mTORC2 integrity and function (Fig. 3). Consistent with this notion, compared to Sin1-WT, relatively sustained Akt phosphorylation was observed in Sin1-R81T expressing cells. At the cellular levels, expressing Sin1-R81T, opposing to the phospho-mimetic Sin1-T86E/T398E mutant, led to comparable levels of cleaved caspase 3 to cells expressing Sin1-WT upon etoposide or cisplatin challenge, subsequently conferring cellular resistance to these agents. More importantly, compared to Sin1-WT, Sin1-R81T expressing cells gained oncogenic ability to promote ovarian cancer cell growth in soft agar as well as in a xenograft mouse model. Together, these data consistently support a model that Sin1 phosphorylation plays critical roles in inhibiting mTORC2 oncogenic function in both physiological and pathological settings.

## Implications of mTORC1-mediated suppression of mTORC2

Compared to the extensively studied regulations of the mTORC1 complex, the upstream regulatory signaling pathways governing mTORC2 activity remained largely to be determined. So far, the only defined upstream regulation of mTORC2 is the requirement for ribosome association for its activation, where the mechanisms remain largely uncharacterized (Zinzalla et al., 2011). As S6K and 4EBP-1, two characterized mTORC1 substrates, are indispensible for translation of capped mRNAs by ribosome, there might be some yet-to-be identified positive regulatory events governed by S6K or 4EBP-1, which are vital for mTORC2 activation on ribosome. Furthermore, given that both mTORC1 and mTOCR2 contain mTOR, it is not surprising that many layers of crosstalks might exist between these two complexes.

Recent studies have begun to reveal a complicated cross-communication between these two mTOR-containing complexes while the exact molecular mechanism(s) remain largely elusive. In this effort, we have defined a novel and independent negative feedback loop through which either S6K1 or Akt directly phosphorylates Sin1 to repress mTORC2 activation in epithelial cells or adipocytes, respectively, providing a possible molecular mechanism for mTORC1 to balance the extra- and intra- cellular signals. Moreover, systematic analyses of many signaling pathways revealed a general oscillation pattern of activation/inactivation signaling dynamics (Purvis et al., 2012; Purvis and Lahav, 2012, 2013). This also applied to mTORC2-mediated growth factor signaling pathways, as suggested by the periodic Akt activation pattern upon stimulation by insulin or growth factors (Purvis and Lahav, 2013). To this end, mTORC1-mediated inhibition of mTORC2 through Sin1 phosphorylation might be one of such mechanisms, in addition to de-phosphorylation of Akt and multiple other negative feedback mechanism, to ensure that mTORC2 is only activated in a “pulse” manner (Chen et al., 2012). Therefore, between these two mTOR-containing complexes, it is plausible that mTORC1 exhibits constant basal activity whereas the mTORC2 complex is only transiently activated following external stimuli. This may partially explain the fact that hyper-activation of the critical signaling pathways including mTORC2/Akt is a hallmark for majority of human cancers (Testa and Tsichlis, 2005).

Finally, the identification of this mTORC1/S6K feedback suppression of mTORC2 expanded the critical role of mTORC1 in regulating and coordinating various growth factor signaling pathways. Compared to the previously identified negative feedback regulatory loops via IRS-1 (Harrington et al., 2004; Shah and Hunter, 2006) or Grb10 (Hsu et al., 2011; Yu et al., 2011), which nicely explained the IGF-1 and insulin but not the PDGF or EGF signaling regulatory pathways, the mTORC1/S6K1-mediated phosphorylation of Sin1 could function to balance the mTORC2/Akt activation in response to a wider range of growth stimuli beyond insulin and IGF-1, including but not limited to PDGF and EGF. Moreover, by targeting the mTORC2-specific component Sin1, instead of IRS-1 or Grb10 that are upstream of both mTORC1 and mTORC2, for phosphorylation and inhibition refines the suppression effects mainly on mTORC2, suggesting that this newly identified mechanism might be a specific mTORC2-targeted negative regulation. Taken together, our study unravels a novel IRS-1/Grb10-independent feedback mechanism of the tightly regulated PI3K/mTORC2/Akt pathway. More significantly, an ovarian cancer patient-derived R81T mutation of Sin1 was demonstrated to gain oncogenic capacity by bypassing this negative regulation due to the lack of Sin1-T86 phosphorylation, providing a molecular mechanism for the elevated mTORC2/Akt activation that could potently promote tumorigenesis at least in this cancer patient. In summary, our work points to the prospect of targeting Sin1 phosphorylation signaling as an effective therapeutic strategy in treating human disorders such as diabetes and cancer.
